# Impact of Fermented Dairy on Gastrointestinal Health and Associated Biomarkers

**DOI:** 10.1093/nutrit/nuaf114

**Published:** 2025-07-24

**Authors:** Glory Bui, Maria L Marco

**Affiliations:** Department of Food Science and Technology, University of California, Davis, CA 95616, United States; Department of Food Science and Technology, University of California, Davis, CA 95616, United States

**Keywords:** yogurt, milk, kefir, gut health, intestinal barrier, gut microbiome, inflammation, probiotics

## Abstract

In this narrative review, we examined observational and randomized controlled trials investigating the effects of fermented dairy foods, including yogurt, fermented milk, kefir, and cheese, on gastrointestinal (GI) symptoms and/or GI biomarkers of health. Studies that recorded GI symptoms such as intestinal discomfort, flatulence, gastroenteritis, diarrhea, and constipation were included. GI health biomarkers encompassed measurements of intestinal integrity or permeability (eg, endotoxemia, zonulin, 2-arachidonoylglycerol), immune responses (eg, TNFα, IL-6, high-sensitivity CRP [hs-CRP], IFNγ, IL-1β, CCL5, TGFβ, IL-10, secretory IgA, α- and β-defensin, and LL-37), fecal microbiota, and fecal short-chain fatty acids (SCFAs). Studies on probiotic-containing fermented dairy foods were included if the primary focus was the fermented dairy food, not specific probiotic strains. Thirty-seven reports met the inclusion criteria and encompassed studies on healthy children, healthy adults, or individuals with underlying conditions. Twenty-one of these studies included fermented dairy products with probiotic strains. No study reported a harmful impact of fermented dairy on gut health. Ten studies reported no benefit of fermented dairy on GI symptoms or immune biomarkers compared with milk or when no dairy was consumed. The remaining studies described significant changes in one or more gut symptoms or biomarkers with fermented dairy intake. Improvements in GI symptoms, such as abdominal pain or discomfort, flatulence, constipation, and IBS severity, were found in most studies for which such symptoms were assessed. Reductions in intestinal inflammatory markers, specifically serum TNFα levels, were found to be associated with fermented dairy intake. In several trials, significant alterations to the gut microbiota or increased levels of fecal SCFAs following fermented dairy intake were measured, but not all of those studies incorporated clinically relevant outcomes. New investigations evaluating the impact of fermented dairy on gut health should build upon the findings of these prior studies, considering target populations, underlying health conditions, and relevant gut health end points.

## INTRODUCTION

The gastrointestinal (GI) tract is responsible for human nutrition via its activities that result in the digestion of foods and absorption of nutrients and other bioactive compounds. The GI tract is also increasingly recognized for health-modulatory facets well beyond nutrition. For example, the GI tract contains the largest number of immune cells in the body and controls inflammatory responses that impact local GI and systemic health.^1^^,^^2^ The intestine also contains diverse cell types that signal to the brain along the gut–brain axis, in ways that impact mood, behavior, and appetite.^3^^,^^4^ In addition to the importance of the intestinal epithelium in guiding these effects, the GI tract also contains over a trillion microbes that have integral roles in all facets of intestinal and bodily function and whose composition is strongly influenced by host health, dietary intake, and other intrinsic and extrinsic factors.^5^^,^^6^ The significant interlinking relationship between the GI tract and human health has led to a definition of GI (or gut) health as a “state of physical and mental well-being in the absence of GI complaints that require the consultation of a doctor, in the absence of indications of or risks for bowel disease and in the absence of confirmed bowel disease.”^7^ As a result, assessing gut (or GI) health requires evaluating symptoms and key biomarkers associated with inflammation, barrier integrity, and metabolism, all of which may be influenced by gut microbiome composition.

Expansion in the use of the “gut health” terminology among consumers and clinicians^8–10^ has led to an increased number of human studies investigating the impact of various diets and individual foods or supplements on gut health.^11–17^ Dietary intake of different foods and nutrients is intrinsically linked to GI function, as specific components can either reduce or promote intestinal transit times, bloating, and other feelings of discomfort, along with effects on satiety or immune responses.^18–21^ For example, a high-fat diet, antibiotics, alcohol, psychological stress, and other environmental and genetic factors are associated with disrupting the gut epithelium, resulting in a low, but sustained, inflammatory response.^22^^,^^23^ Conversely, Mediterranean diets and diets containing high levels of fiber improve gut health by reducing inflammatory markers.^18^^,^^24–26^ Therefore, understanding individual foods and their roles in gut health is expected to improve overall dietary recommendations.

The fermentation of milk by lactic acid bacteria and other microorganisms yields various dairy products (eg, yogurt, kefir, fermented milk, and cheese). Randomized controlled trials (RCTs) and observational studies of fermented dairy product consumption have shown that these foods can improve weight maintenance and cardiovascular health and reduce the risk of type 2 diabetes mellitus.^27–29^ Fermented dairy foods are also the most common delivery format of probiotics to the GI tract.^30^^,^^31^ The use of dairy for probiotic delivery may be beneficial, because animal and cell culture studies suggest that including dairy could improve probiotic survival and efficacy in situ.^32–34^ However, not all strains in fermented dairy foods have the probiotic designation. For example, *Streptococcus thermophilus* and *Lactobacillus delbrueckii* subspecies *bulgaricus* are needed to make yogurt, but commercial yogurts frequently contain added probiotic strains validated for other health benefits unrelated to the milk substrate. Therefore, some, but not all, fermented dairy foods will have verified probiotic strains intended to confer specific health benefits.

In this narrative review, we examined human studies on yogurt, fermented milk, kefir, and cheese which measured clinical symptoms and molecular biomarkers associated with gut health. The rationale for examining the literature in this way is that the impacts of fermented dairy under the “gut health” framework have yet to be holistically examined. Metrics in these trials included the assessment of GI symptoms, function, and motility,^35^^,^^36^ biomarkers of gut barrier integrity and mucosal immunity, as well as the gut microbiome composition.^37–39^ Studies on probiotic-fermented or probiotic-containing fermented dairy products were included if the focus was on the effect of the fermented milk and not the fermented milk with and without the probiotic strain. Sources of variability between study outcomes and future research directions necessary for understanding the effects of fermented dairy foods on gut health are discussed.

## METHODS

The literature searches included all years up to July 2024 using PubMed (https://pubmed.ncbi.nlm.nih.gov/) and Web of Science (https://www.webofscience.com). For the literature searches, no restrictions were placed on participant age or where the study was performed. Key search terms included “gut symptoms” OR “GI symptoms” OR “gut well-being” OR “inflammatory bowel syndrome” OR “IBS” OR “inflammatory bowel disease” OR “IBD” OR “abdominal dysfunction” OR “intestinal barrier” OR “e-cadherin” OR “growth factors” OR “tight junction molecules” OR “antitrypsin” OR “LPS” OR “alpha defensin” OR “beta defensin” OR “calprotectin” OR “lysozyme” OR “elastase” OR “inflammatory markers” OR “inflammation” OR “inflammatory cytokine” OR “short chain fatty acids” OR “zonulin” OR “gut motility” OR “metabolome” OR “gut permeability” OR “gut mucosa” OR “calprotectin” OR “S100A12” OR “lactoferrin” OR “M2-PK” OR “neopterin” OR “metalloproteinases” OR “myeloperoxidases” OR “MPO” OR “polymorphonuclear elastase” OR “a1-antitrypsin” OR “CH13L1” OR “NGAL” OR “PMN-E” OR “a1-acid glycoprotein” OR “b2-microglobulin” OR “adenosine deaminase” OR “CRP” OR “DPP-4” OR “CpG” or “NGAL-MMP9” OR “procalcitonin” OR “serum amyloid A” OR “sialic acid” OR “TFF3” OR “tryptophan” AND “fermented milk” OR “yogurt” OR “kefir” OR “cultured milk products” AND “clinical trial” OR “randomized controlled trial.” Only full-text articles written in English of completed human trials assessing bovine dairy products were included. Animal and in vitro studies were excluded. Various study types were included, encompassing randomized controlled trials (RCTs), either with a parallel group (PG-RCT), crossover (CO-RCT), or block design, cross-sectional analyses, open-design studies, and open prospective controlled studies.

## OVERVIEW OF FERMENTED DAIRY STUDIES EXAMINING GUT HEALTH

Based on the literature search and inclusion criteria, a total of 37 human studies were examined further in this review (Table 1). Among them, 26 were PG-RCTs, 6 were CO-RCTs, 2 were cross-sectional studies, 1 was a block design RCT, 1 was an open-design study, and 1 was an open prospective controlled study. In total, 15 studies were placebo-controlled (PC). Among the RCTs, the studies included 13 to 391 subjects, with ages ranging from 3 months to 76 years. For the cross-sectional studies, the minimum number of subjects was 130, and the maximum number was 3042, with ages ranging from 18 to 89 years. The effects of fermented dairy were assessed (i) relative to baseline levels, (ii) with exclusion/reduction of dairy from the diet, (iii) relative to milk intake, and/or (iv) compared with a nondairy, energy-matching product. The fermented dairy products used encompassed yogurt (12 studies), fermented milk (17 studies), kefir (3 studies), and cheese (1 study). Additionally, 1 study assessed plain and flavored kefir and yogurt compared with milk, 1 study assessed both yogurt and fermented milk, and 2 studies assessed multiple fermented dairy types (eg, yogurt, fermented milk, and cheese). Probiotic strains were named in 21 of the studies, including 5 of the 14 trials on yogurt and 16 of the 17 trials in which fermented milk was examined. These probiotic strains were *Bifidobacterium lactis* DN-173 010 (also named CNCM I-2494) (7 studies), *B bifidum* YIT 4007 (1 study), *Bifidobacterium animalis* subsp. lactis BB-12 (1 study), *Lacticaseibacillus paracasei* Shirota YIT 9029, (2 studies), *L paracasei* CBA L74 (3 studies), *L rhamnosus* GG (1 study), *L casei* DN-114 001 (1 study), a mixture of *Lactobacillus acidophilus* La5 and *B lactis* BB-12 (2 studies), a mixture of *L paracasei* F19, *Lactobacillus acidophilus* La5 and *B lactis* BB-12 (2 studies), or a mixture of *L paracasei* CNCM I-1518, *L paracasei* I-3689 and *L rhamnosus* CNCM I-3690 (1 study). Six others may have contained probiotics, but the names of the specific microbial strains present were not provided, rendering them ineligible for probiotic classification, despite using the term probiotic in publication.

**Table 1.** Human Studies Assessing the Impact of Fermented Dairy Consumption on Gut Symptoms and Biomarkers for Gut Health

Study	Trial type	Duration	Population	Treatment group		Control group	Gut Sx	Biomarkers for gut health			
				Fermented dairy type	Probiotic strain^a^			Immunity	Barrier integrity	Gut microbiota	SCFAs
Guyonnet et al (2009)^42^	DB-PG-RCT	4 weeks	Women with minor digestive symptoms	Fermented milk	*B* *lactis* DN-173 010	Milk	**✓**	ND	ND	ND	ND
Kato-Kataoka et al (2016)^43^	PC-DB-PG-RCT	8 weeks	Healthy medical students with stress-induced abdominal dysfunction	Fermented milk	*L* *paracasei* YIT 9029	Milk	**✓**	ND	ND	**✓**	ND
Hertzler et al (2003)^44^	BD-RCT	8 hours	Healthy adults with lactose maldigestion	Plain/flavored kefir^b^ or plain/flavored yogurt	ND	Milk	**✓**	ND	ND	ND	ND
Le Nevé et al (2020)^45^	ODS	28 days	Healthy adults given a high-residue diet	Fermented milk	*B* *lactis* DN-173 010^f^	No control group	**✓**	ND	ND	**X**	ND
Yang et al (2008)^46^	PC-PG-RCT	2 weeks	Adult women with constipation	Fermented milk	*B* *lactis* DN-173 010	Milk	**✓**	ND	ND	ND	ND
Zhang et al (2021)^47^	PC-DB-PG-RCT	9 weeks	Patients with depression (on depressive regimens), specific etiology, and gut microbiota	Fermented milk	*L* *paracasei* YIT 9029	Milk	**✓**	**✓**	ND	**✓**	ND
Miki et al (2007)^50^	PC-DB-PG-RCT	12 weeks	Adult patients	Fermented milk	*B* *bifidum* YIT 4007	Milk	**✓**	ND	ND	ND	ND
Guillemard et al (2021)^51^	PC-DB-PG-RCT	28 days	Adult patients undergoing *H* *pylori* eradication therapy	Fermented milk	*L* *paracasei* CNCM I-1518, *L* *paracasei* CNCM I-3689, *L* *rhamnosus* CNCM I-3690	Milk	**X**	**O**	ND	**✓**	**✓**
Liu et al (2010)^54^	PG-PP-RCT	14 days	Adults with chronic liver disease	Yogurt^b^	ND	No yogurt	**✓**	ND	ND	**✓** ^h^	ND
Agrawal et al (2009)^56^	PC-DB-PG-RCT	4 weeks	Female patients with constipation-predominant IBS	Fermented milk	*B* *lactis* DN-173 010	Milk	**✓**	ND	ND	ND	ND
Zeng et al (2008)^57^	PC-SB-PG-RCT	4 weeks	Adults with diarrhea-predominant IBS	Fermented milk^b^	ND	Milk	**✓**	ND	**✓**	ND	ND
Le Nevé et al (2019)^58^	PC-DB-PG-RCT	14 days	Adult IBS patients given a nutrient and lactulose challenge	Fermented milk	*B* *lactis* CNCM I-2494^f^	Milk	**✓**	ND	ND	**✓**	ND
Søndergaard et al (2011)^59^	PC-DB-PG-RCT	8 weeks	Adult IBS patients	Fermented milk	*L* *paracasei* F19, *L* *acidophilus* La5, *B* *lactis* BB-12	Milk	**O**	ND	ND	ND	ND
Simrén et al (2010)^60^	PC-DB-PG-RCT	8 weeks	Adult IBS patients	Fermented milk	*L* *paracasei* F19, *L* *acidophilus* La5, *B* *lactis* BB-12	Milk	**O**	ND	ND	ND	ND
Roberts et al (2013)^61^	DB-PG-RCT	4-12 weeks	Adult IBS patients	Yogurt	*B* *lactis* CNCM I-2494^f^	Milk	**X**	ND	ND	ND	ND
Tabbers et al (2011)^62^	PC-DB-PG-RCT	3 weeks	Children (3–16 years) with constipation	Fermented milk	*B* *lactis* DN-173 010	Milk	**X**	ND	ND	ND	ND
Bhatnagar et al (1998)^64^	PG-RCT	≥3 days	Malnourished male children (4-48 months)	Yogurt	ND	Milk	**X**	ND	ND	ND	ND
Boudraa et al (1990)^65^	PG-RCT	5 days	Children with persistent diarrhea	Yogurt	ND	Milk	**✓**	ND	ND	ND	ND
Corsello et al (2017)^66^	PC-DB-PG-RCT	3 months	Healthy children (12-48 months)	Fermented milk*	*L* *paracasei* CBA L74	Nondairy product^g^	**✓**	**✓**	ND	ND	ND
Nocerino et al (2017)^67^	PC-DB-PG-RCT	3 months	Healthy children (12-48 months)	Fermented milk*	*L* *paracasei* CBA L74	Nondairy product^g^	**✓**	**✓**	ND	ND	ND
Wang et al (2004)^68^	PG-RCT	6 weeks	Adult patients undergoing *H* *pylori* eradication therapy	Yogurt	*L* *acidophilus* La5*, B* *lactis* Bb12	Milk	ND	**✓**	ND	ND	ND
Sheu et al (2006)^69^	PG-RCT	4 weeks	Adult patients undergoing *H* *pylori* eradication therapy	Yogurt	*L* *acidophilus*, La5*, B* *lactis* Bb12	No yogurt	ND	**✓**	ND	ND	ND
Panagiotakos et al (2010)^77^	CSS	NA	Healthy adults	Moderate or frequent fermented dairy intake per week^c^	ND	Rare or very rare fermented dairy intake per week^c^	ND	**✓**	ND	ND	ND
González et al (2019)^78^	CSS	NA	Healthy adults	Fermented dairy product within a regular diet	ND	Nonconsumers of specified dairy product	ND	**✓**	ND	**✓** ^h^	**✓**
Chen et al (2019)^79^	PG-RCT	24 weeks	Adult obese women with both nonalcoholic fatty liver disease and metabolic syndrome	Yogurt	ND	Milk	ND	**✓**	**✓**	**✓**	ND
Bellikci-Koyu et al (2019)^80^	PC-PG-RCT	12 weeks	Adults with metabolic syndrome	Kefir^b^	ND	Milk	ND	**✓**	ND	**✓**	ND
Burton et al (2017)^81^	DB-CO-RCT	2 weeks	Healthy young men	Yogurt^a^	*L* *rhamnosus* GG	Milk	ND	**O**	ND	**✓**	ND
Labontè et al (2014)^82^	SB-CO-RCT	4 weeks	Healthy adults with high-sensitivity CRP values of >1 mg/L	Dairy products^d^	ND	Nondairy product^g^	ND	**O**	ND	ND	ND
Sandby et al (2024)^83^	PG-RCT	16 weeks	Adult males with abdominal obesity	Yogurt or heat-treated yogurt	ND	Milk or acidified milk	ND	**X**	ND	ND	ND
McKinlay et al (2022)^84^	DB-CO-RCT	2 × 5-day sessions	Healthy female adolescents	Yogurt	ND	Nondairy product^g^	ND	**✓**	ND	ND	ND
Pei et al (2017)^85^	PG-RCT	9 weeks	Premenopausal healthy and obese adult women	Low-fat yogurt	ND	Soy pudding (nondairy)	ND	**✓**	**✓**	ND	ND
Pražnikar et al (2020)^91^	CO-RCT	3 weeks	Asymptomatic overweight adults	Kefir^b^	ND	Milk	ND	ND	**✓**	ND	ND
Volokh et al (2019)^104^	OPCS	30 days	Healthy adults	Yogurt	*B* *lactis* BB-12	No control group	ND	ND	ND	**✓**	ND
Cannavale et al (2022)^105^	SB-CO-RCT	4 weeks	Healthy adults	Kefir^b^	ND	Milk	ND	ND	ND	**✓**	ND
Guerin-Danan et al (1998)^107^	PG-RCT	1 month	Infants (10-18 months)	Yogurt or fermented milk^e^	*L* *casei* DN-114 001^e^	Milk	ND	ND	ND	**✓** ^h^	**✓**
Canani et al (2017)^108^	PC-DB-PG-RCT	3 months	Healthy children (1-2 years)	Fermented milk	*L* *paracasei* CBA L74	Nondairy product^g^	ND	ND	ND	**✓**	**✓**
Zheng et al (2015)^106^	CO-RCT	3 × 2 weeks	Healthy adults	Cheese	ND	Milk	ND	ND	ND	ND	**✓**

a Product may contain additional nonprobiotic microbes, including yogurt starters.

b Might contain probiotic strain(s).

c Moderate is 11-14 servings; frequent is >14 servings; rare is 8–11 servings; very rare is <8 servings.

d Dairy products included low-fat milk, low-fat yogurt, and regular-fat cheddar cheese.

e Probiotic is only included in fermented milk.

f Strain DN-173 010 is also known as CNCM I-2494.

g Nondairy product with similar energy content to that of the fermented dairy product.

h Measured selected microbes via quantitative RT-PCR or in laboratory culture medium. Abbreviations: **✓**, some symptoms or biomarker significantly changed; **O**, no significant difference after fermented dairy consumption compared with control group; **X**, no significant changes compared with baseline or control group; *B* *bifidum*, *Bifidobacterium*  *bifidum*; BD, block design; *B* *lactis*, Bifidobacterium animalis subsp. lactis; CO, crossover; CSS, cross-sectional study; DB, double-blind; *H* *pylori*, *Helicobacter pylori*; *L* *acidophilus*, *Lactobacillus acidophilus*; *L* *casei*, *Lactobacillus casei*; *L* *paracasei*, *Lactobacillus paracasei*; *L* *rhamnosus*, *Lactobacillus rhamnosus*; NA, not applicable; ND, not described; ODS, open design study; OPCS, open prospective controlled study; PC, placebo-controlled; PG, parallel-group; PP, pre-test–post-test; RCT, randomized controlled trial; SB, single-blind; SCFA, short chain fatty acid; Sx, symptoms.

## EFFECTS OF FERMENTED DAIRY ON GI SYMPTOMS

Of the 37 studies examined, 20 measured GI symptoms, either through patient complaints or clinical monitoring (Table 1). Assessment of GI symptoms included patient symptom surveys^40^ or diagnostic testing of breath hydrogen levels, the latter of which serves as a biomarker for malabsorption of carbohydrates linked to GI symptoms, including flatulence, bloating, diarrhea, and abdominal pain.^41^ The following sections discuss the findings for how fermented dairy consumption affected the intestinal symptoms of healthy adults, adults with underlying conditions (*Helicobacter pylori* infection, chronic liver disease, or inflammatory bowel syndrome [IBS]), and children (Table 2).

**Table 2.** Gastrointestinal Symptoms and Biomarkers, and Studies Showing Significant Changes in Gut Health Metrics with Fermented Dairy Consumption

	Compared with baseline levels		Compared with diet or control that excluded dairy		Compared with milk consumption	
	↑	↓	↑	↓	↑	↓
Gastrointestinal symptoms						
Overall GI well-being			Le Nevé et al (2020)^45^		Guyonnet et al (2009)^42^	
Colonic transit or stool frequency		Yang et al (2008)^46^			Yang et al (2008)^46^, Agrawal et al (2009)^56^	
Appetite and food intake	Liu et al (2010)^54^		Liu et al (2010)^54^			
GI symptoms^a^ or discomfort						Kato-Kataoka et al (2016)^43^, Miki et al (2007)^50^, Le Nevé et al (2019)^58^
Acute gastroenteritis				Corsello et al (2017)^66^, Nocerino et al (2017)^67^		
IBS severity/score^†^		Zeng et al (2008)^57^, Søndergaard et al (2011)^59^, Simrén et al (2010)^60^				Agrawal et al (2009)^56^
Ascites, abdominal distension or dysfunction^c^		Liu et al (2010)^54^, Zeng et al (2008)^57^		Liu et al (2010)^54^		Kato-Kataoka et al (2016)^43^, Agrawal et al (2009)^56^
Borborygmi frequency						Guyonnet et al (2009)^42^
Flatulence frequency or sensation		Le Nevé et al (2020)^45^, Zeng et al (2008)^57^				Guyonnet et al (2009)^42^, Hertzler et al (2003)^44^
Stool consistency		Yang et al (2008)^46^				Guyonnet et al (2009)^42^, Yang et al (2008)^46^
Constipation or defecation difficulty		Yang et al (2008)^46^				Yang et al (2008)^46^, Zhang et al (2021)^47^
Rectal tearing and bleeding						Zhang et al (2021)^47^
Persistent diarrhea^e^						Boudraa et al (1990)^65^
Biomarkers of proinflammatory immune response						
*H pylori* density		Wang et al (2004)^68^, Sheu et al (2006)^69^				
Gastritis activity		Wang et al (2004)^68^				
TNFα		Zhang et al (2021)^47^, Bellikci-Koyu et al (2019)^80^, Burton et al (2017)^81^, Pei et al (2017)^85^		Panagiotakos et al (2010)^77^		Chen et al (2019)^79^
IL-6		Zhang et al (2021)^47^, Burton et al (2017)^81^, Labontè et al (2014)^82^		Panagiotakos et al (2010)^77^		Zhang et al (2021)^47^
CRP or hs-CRP				Panagiotakos et al (2010)^77^, González et al (2019)^78^		
IL-1β		Zhang et al (2021)^47^				
IFNγ		Bellikci-Koyu et al (2019)^80^				
Biomarkers of anti-inflammatory immune response						
Secretory IgA			Corsello et al (2017)^66^, Nocerino et al (2017)^67^			
α- and β-defensin			Corsello et al (2017)^66^, Nocerino et al (2017)^67^			
LL-37			Corsello et al (2017)^66^, Nocerino et al (2017)^67^			
CCL5		Burton et al (2017)^81^				
IL-10			McKinlay et al (2022)^84^			
TGFβ^d^			Pei et al (2017)^85^			
Biomarkers of intestinal integrity						
Lactulose/mannitol		Zeng et al (2008)^57^				
LPS						Chen et al (2019)^79^
LBP/sCD14				Pei et al (2017)^85^		
IgM EndoCAb	Pei et al (2017)^85^					
2-arachidonoylglycerol			Pei et al (2017)^85^			
Zonulin						Pražnikar et al (2020)^91^

a Gastrointestinal symptoms defined as acid reflux syndrome/pyrosis, abdominal pain syndrome/gastric pain, indigestion syndrome/disgust/stomach complaint, diarrhea syndrome, constipation syndrome, nausea, and/or belch.

b IBS scores included assessments of abdominal pain, reflux, diarrhea, indigestion, and constipation.

c Abdominal dysfunction as defined as “abdominal discomfort and pain, the feeling of incomplete evacuation, abdominal distention, straining during bowel movement, and gastric pain.”

d Expression level in PBMCs.

e Defined as the clinical failure rate for treating persistent diarrhea. Abbreviations: ↑, significant increase; ↓, significant decrease; hs-CRP, high-sensitivity CRP; IBS, irritable bowel syndrome.

### Fermented Dairy and GI Symptoms of Healthy Adults

Two of the studies in which fermented dairy was consumed by healthy individuals found improvements in GI symptoms, including abdominal pain, flatulence, stool frequency and consistency, and bloating.^42^^,^^43^ In a double-blind (DB) PG-RCT study that assessed women with minor digestive disorders (*N* = 197 subjects), consumption of fermented milk containing probiotic *B lactis* DN-173 010 along with yogurt starter cultures (*S thermophilus* CNCM I-1630, *L delbrueckii* subspecies *bulgaricus* CNCM I-1632 and I-1519), and *Lactococcus cremoris* CNCM I-1631 for 4 weeks improved overall GI well-being and quality of life compared with a control group given milk.^42^ Based on questionnaire responses, fermented milk intake was associated with a lower composite score of digestive symptoms, including a reduced frequency of borborygmi and flatulence. Stool consistency scores were also significantly decreased (ie, became more normal) after probiotic fermented milk consumption compared with milk consumption.^42^ After a 4-week washout period, there were no significant differences between the 2 groups. In the other study, milk intake was compared with that of fermented milk containing the probiotic *L casei* Shirota YIT 9029 in an 8-week, PC-DB-PG-RCT with healthy medical students (*N* = 47 subjects).^43^ Outcomes indicated that there were fewer stress-induced abdominal dysfunctions and GI symptoms associated with the fermented milk intake. Abdominal dysfunction was defined by the severity of 5 common symptoms: “abdominal discomfort and pain,” “feeling of incomplete evacuation,” “abdominal distention,” “straining during bowel movement,” and “gastric pain.” Symptoms were assessed using a GI symptom rating scale.^43^

Another 2 studies performed with healthy adults assessed whether the consumption of fermented dairy could help alleviate diet-induced intestinal discomfort. Adults with lactose maldigestion in an 8-hour randomized, controlled block design trial (*N* = 15 subjects) had improved lactose digestion and tolerance after consuming either kefir or yogurt (bacterial species and strain designations were not provided for either product type), compared with milk.^44^ Both kefir and yogurt were associated with reduced flatulence severity and breath hydrogen levels. The other report was on an open-design, 3-day, high-residue diet study (*N* = 63 subjects).^45^ Subjects eating a plant-based diet rich in fermentable residues (ie, a high-residue diet) reported reductions in gas-related symptoms and number of daily gas evacuations after consuming fermented milk containing the probiotic, *B lactis* CNCM I-2494, and other strains, namely *S thermophilus* CNCM I-2773, CNCM I-2130, and CNCM I-2272, *Lactobacillus delbrueckii* subspecies *bulgaricus* CNCM I-1519, and *Lactococcus lactis* subspecies *lactis* CNCM I-1631. This was quantified based on daily symptom questionnaire responses of well-being and the number of daytime anal gas evacuations compared with baseline values.^45^

Two studies with healthy adults examined the effects of fermented dairy foods on constipation.^46^^,^^47^ Constipation-associated symptoms encompass abdominal and rectal discomfort, which may lead to sensations of rectal burning, tearing, or bleeding due to the presence of hardened stools.^48^^,^^49^ In a PC-PG-RCT (*N* = 126 subjects), fermented milk containing probiotic *B lactis* DN-173 010 and yogurt starter strains (*S thermophilus* and *L delbrueckii* subspecies *bulgaricu*s) for 2 weeks by women with constipation increased stool frequency, decreased defecation difficulty (constipation), and improved stool consistency compared with baseline levels and with those who drank milk.^46^ In a PC-DB-PG-RCT (*N* = 69 subjects), the consumption of fermented milk containing probiotic *L* *casei* Shirota strain YIT 9029 for 9 weeks by patients experiencing depression resulted in significant differences in total patient constipation-symptom scores compared with patients drinking a placebo milk.^47^ Specific symptoms, including stool symptoms and rectal tearing or bleeding, were also significantly reduced after fermented milk consumption compared with the milk placebo group.

### Fermented Milk and GI Symptoms in Adults with Underlying Conditions

Fermented milk containing *S thermophilus* YIT 2021 and the putative probiotic strain *B bifidum* YIT 4007 improved the rate of upper GI symptom relief reported in physician interviews.^50^ This 12-week PC-DB-PG-RCT (*N* = 79 subjects) with a 12-week product-ingestion period also found that the majority (*n* = 69) of study participants had Urea Breath Test (UBT) levels indicative of *H pylori* infection. Examination of changes in UBT and serum pepsinogen (PG) indicated that consuming fermented milk with *B bifidum* YIT 4007 may have suppressed *H pylori* activity.^50^ However, in another study, consumption of a multistrain fermented milk (including probiotic *L paracasei* CNCM I-1518 and CNCM I-3689, *L rhamnosus* CNCMI-3690, and 4 other strains used to make the fermented product) for 28 days in a DB-PG-PC-RCT (*N* = 136 subjects) did not alter GI symptoms or antibiotic-associated diarrhea among patients undergoing *H pylori* treatment.^51^

Chronic liver disease is associated with intestinal distension, delayed gut transit, and ascites, which can lead to reduced food intake.^52^^,^^53^ In a pre-test–post-test PG-RCT (*N* = 81 subjects), chronic liver disease patients reported that the consumption of yogurt containing “Bacillus bifidus” (not a verified bacterial species)*, L acidophilus, L delbrueckii* subspecies *bulgaricus,* and *S thermophilus* (strain designations not provided) for 14 days resulted in an increase in appetite and food intake and decreased abdominal distension and hypodynamia, compared with patients who did not consume any yogurt.^54^

IBS symptoms include abdominal pain or discomfort and irregular bowel habits that may confer predominant episodes of constipation (IBS-C), diarrhea (IBS-D), or a mixture of both.^55^ A total of 6 studies thus far have examined the potential of fermented dairy foods to alleviate IBS symptoms. For patients with IBS-C, consumption of fermented milk containing probiotic *B lactis* DN-173 010 and yogurt starters (*S thermophilus* and *L delbrueckii* subspecies *bulgaricus*), as opposed to milk, was found to reduce mouth-to-cecum and colonic transit times and reduce overall IBS severity, abdominal pain/discomfort, and the percentage change in maximal abdominal distension (9-week, PC-DB-PG-RCT, *N* = 34 subjects).^56^ In a single-blind PC-PG-RCT with IBS-D patients (*N* = 30 subjects), consumption of fermented milk (containing undefined strains of *S thermophilus*, *L delbrueckii* subspecies *bulgaricus*, *L acidophilus*, and *Bifidobacterium longum*) for 4 weeks reduced abdominal pain, sensation of flatulence, and IBS symptoms as assessed by the Gastrointestinal Symptom Rating Scale (GSRS) questionnaire, compared with baseline levels, whereas milk intake did not result in the same benefit.^57^ In a study assessing all IBS subtypes, patients who had elevated breath H_2_ levels at baseline and consumed fermented milk containing probiotic *B lactis* CNCM I-2494 (together with *S thermophilus* CNCM I-163, *L delbrueckii* subspecies *bulgaricus* CNCM I-1632 and CNCM I-1519, and *Lactococcus lactis* subspecies *lactis* CNCM I-1631) twice a day for 14 days had a trending but nonsignificant (*P* = .05) reduction in GI discomfort compared with those who consumed milk (PC-DB-PG-RCT, *N* = 106 subjects).^58^ These patients also experienced decreased fasting H_2_ levels and H_2_ production after consuming the probiotic fermented milk, compared with the milk control group, suggesting improved carbohydrate absorption.^58^ Additionally, in 2 PC-DB-PG-RCT studies (*N* = 52 and *N* = 74 subjects), intake of acidified milk or a commercially prepared fermented milk (Cultura; including probiotic strains *L *paracasei F19, *L acidophilus* La5, and *B lactis* BB-12 as well as *S thermophilus* and *L bulgaricus*), resulted in similarly improved IBS symptoms after 8 weeks compared with baseline levels, but the improvements were similar for patients given milk.^59^^,^^60^ Lastly, a DB-PG-RCT study found that consumption of either probiotic yogurt (*B lactis* I-2494 [DN-173 010], *S thermophilus* I-1630, and *L delbrueckii* subspecies *bulgaricus* I-1632 and I-1519) or milk (adjusted for lactose content) for up to 12 weeks did not improve symptoms in IBS patients with constipation (IBS-C or mixed profile) (*N* = 76 subjects).^61^

### Fermented Dairy and GI Symptoms in Children

A prospective PC-DB-PG-RCT trial with constipated children (*N* = 74 subjects) found no improvement in stool frequency between participants consuming milk and those consuming fermented milk containing probiotic *B lactis* DN-173 010 and the yogurt cultures *S thermophilus* CNCM I-1630, *L delbrueckii* subspecies *bulgaricus* CNCM I-1632 and I-1519, and *L cremoris* CNCM I-1631.^62^ Previous studies have reported differences in constipation symptoms across multiple age groups,^63^ but it is still unclear whether fermented dairy is more beneficial among adult or pediatric populations.

The capacity of fermented dairy to alleviate diarrhea was evaluated in 2 pediatric studies relevant for inclusion in this review. Substituting yogurt formula with *S thermophilus* and *L delbrueckii* subspecies *bulgaricus* for milk formula in the diet of malnourished children between 4 and 47 months old with acute diarrhea for 3 days did not lead to significant improvements, as determined by stool weights, diarrhea duration, and treatment failure rates in a PG-RCT (*N* = 96 subjects).^64^ Instead, milk intakes were higher than yogurt intakes, and children consuming milk had a higher median percentage weight gain. However, another nonblinded study performed with children of a similar age (3 to 36 months) but with persistent diarrhea reported that consumption of yogurt with *S thermophilus* and *L bulgaricus* over 5 days significantly reduced the clinical failure rate compared with children who consumed milk (*N* = 45 subjects).^65^ Clinical failure was determined if the child lost 5% of their body weight in 24 hours, had no gain in body weight in 3 consecutive days, or if diarrhea persisted through the final day of the study.^65^ The differences between the outcomes of these 2 studies are likely due to a combination of factors, including variations in the patient populations, severity of diarrhea symptoms, and the measured end points.

The capacity of fermented dairy foods to alleviate GI symptoms in children aged 12 to 48 months with chronic infectious diseases (CIDs) was investigated in 2 studies.^66^^,^^67^ Pediatric CIDs are defined as illnesses commonly experienced by children attending daycare or preschool, such as acute gastroenteritis (ie, presence of more than 3 diarrheal bowel movements in 24 hours) and upper respiratory tract infections.^66^^,^^67^ In 2 similar PC-DB-PG-RCT trials (*N* = 126 and *N* = 377), consumption of a fermented milk product containing probiotic *L paracasei* CBA L74 led to a significantly reduced incidence of acute gastroenteritis among children experiencing CIDs compared with children who consumed a maltodextrin drink (nondairy) with a similar energy content.^66^^,^^67^

## EFFECTS OF FERMENTED DAIRY ON GI HEALTH BIOMARKERS

In addition to or instead of measuring GI symptoms using surveys and clinical metrics, human studies examining fermented dairy foods have also assessed gut health using biomarkers (Table 1). As discussed below, a total of 24 studies used biomarkers to measure intestinal inflammatory status, barrier integrity, and/or fecal microbiota and SCFAs (Table 2).

### Fermented Dairy and *H pylori* Infection

Two studies assessed the effect of probiotic yogurt on *H pylori* infection as determined by histological analysis. In an intervention study (*N* = 70 subjects), consumption of yogurt (*S  thermophilus* and *L delbrueckii* subspecies *bulgaricus*) with probiotic *L acidophilus* La5 and *B lactis* BB-12, but not milk, for 6 weeks significantly lowered carbon-13 UBT (C-UBT) values compared with baseline in asymptomatic adults.^68^ From the yogurt group of this study, 14 participants were randomly selected for further follow-up at weeks 12 and 16. Analysis of antral biopsies revealed a significant decrease in *H pylori* density and gastritis activity compared with baseline measurements, but no changes were observed in the gastric body.^68^ This same probiotic yogurt was used in an RCT (*N* = 138) as a 4-week pretreatment to quadruple therapy with patients histologically positive for residual *H pylori* infection.^69^ Consumption of the probiotic yogurt with quadruple therapy significantly decreased the excessive δCO_2_/mL values (an indirect measurement of intragastric *H pylori* loads) of C-UBT after 4 weeks and increased the eradication rate for residual *H pylori* compared with those who only received quadruple therapy.

### Fermented Dairy and Proinflammatory Immune Biomarkers

Milk and dairy products were found in some RCTs to reduce serum inflammatory biomarkers, including CRP, also commonly reported as high-sensitivity CRP (hs-CRP)), cytokines/chemokines (IL-1β, IL-6, IL-8, TNF-α, CCL2, and CCL5), and endotoxin (ie, lipopolysaccharide [LPS]).^70^ These serum immune biomarkers were previously reported to be associated with impaired intestinal barrier integrity and GI symptoms.^71–76^ Therefore, we looked for measurements of these biomarkers in human studies examining fermented dairy foods. A total of 9 out of the 37 studies reported serum biomarkers associated with proinflammatory responses, namely TNFα, IL-6, CRP or hs-CRP, IFNγ, IL-1β, and chemokine ligand 5 (CCL5) (Table 2).

Among these studies, 2 were cross-sectional conducted to examine the impact of fermented dairy consumption relative to individuals who did not consume, or only rarely consumed, fermented dairy.^77^^,^^78^ One of the trials found that healthy adults who consumed fermented dairy products either frequently (>14 servings/week) or moderately (11 to 14 servings/week) had significantly lower levels of serum CRP, IL-6, and TNFα compared with those who consumed <8 servings/week (*N* = 3042 subjects).^77^ The other cross-sectional study (*N* = 130 subjects) assessed healthy individuals who generally ate fermented foods, encompassing 26 different fermented dairy products.^78^ Quantities of serum CRP (the only proinflammatory biomarker examined) were significantly decreased in those who consumed yogurt daily, compared with yogurt nonconsumers.^78^

Reductions in at least one serum proinflammatory biomarker were found in several RCTs on fermented milk. The PC-DB-PG-RCT in which women with IBS-C were examined for GI symptoms after consuming *L paracasei* Shirota YIT 9029 fermented milk also found that those women had significantly reduced quantities of serum IL-6 compared with baseline levels and with those who consumed the milk placebo (Table 1).^47^ However, this study also found that both milk and probiotic fermented milk with *L casei* Shirota strain YIT 9029 were similarly effective in reducing serum TNFα and IL-1β, compared with baseline levels.^47^ In another PG-RCT study, it was found that women with metabolic syndrome (MetS), non-alcoholic fatty liver disease (NAFLD), and obesity (*N* = 92 subjects) had significant decreases in average levels of serum TNFα after consumption of conventional yogurt compared with milk for 24 weeks, but no differences in serum IL-6, IL-1β, or CRP levels were found.^79^

Five other studies found that fermented dairy products reduced biomarkers of inflammation to a similar extent as the controls, or otherwise had no measurable effect. Consumption of either kefir or milk by adults with MetS in a PC-PG-RCT (*N* = 22 subjects) was associated with significantly reduced serum TNFα and IFNγ by the end of the 12-week study.^80^ Interestingly, subjects consuming milk had reduced serum IL-6 levels, but this was not detected for those who drank kefir.^80^ No other changes in inflammation-related biomarkers were found, including hs-CRP, IL-10, alanine aminotransferase, aspartate aminotransferase, or gamma-glutamyl transferase levels.^80^ In a DB-CO-RCT (*N* = 14 subjects) assessing postprandial inflammation caused by a high-fat meal challenge, the consumption of either yogurt (with *S thermophilus* and *L delbrueckii* subspecies *bulgaricus*) containing probiotic *L rhamnosus* GG or milk (acidified with D-(+)-glucono-δ-lactone to the same pH and texture as yogurt) for 2 weeks by healthy men was associated with reduced quantities of serum TNFα, IL-6, and CCL5, compared with baseline levels.^81^ In another RCT (*N* = 112 subjects), adults with elevated baseline hs-CRP levels (>1 and <10 mg/L) either consumed a diet containing fermented/nonfermented dairy foods (milk, yogurt, and cheese) or a nondairy, energy-matched control diet for 4 weeks.^82^ Both diets resulted in similarly reduced serum IL-6 levels, but only the nondairy diet was associated with lower hs-CRP after the intervention.^82^ Notably, this study did not distinguish between fermented dairy versus milk. In the PC-DB-PG-RCT performed over 4 weeks with young and middle-aged adults undergoing *H pylori* eradication treatment (*N* = 136 subjects) (Table 1), twice-daily consumption of a probiotic fermented milk product containing *L paracasei* CNCM I-1518 and I-3689 and *L rhamnosus* CNCM I-3690 (and 4 other *S thermophilus* and *L delbrueckii* subspecies *bulgaricus* strains used to make the fermented product) had no effect on fecal calprotectin, with levels only temporarily increasing in both groups before declining again.^51^ Lastly, in a PG-RCT with 4 arms, adult males with abdominal obesity (*N* = 80) administered yogurt, heat-treated yogurt, milk, or chemically acidified milk over 16 weeks resulted in no significant differences in anthropometry, body composition, or inflammatory markers (CRP, IL-6, and TNFα) between groups.^83^ However, the consumption of yogurt or heat-treated yogurt did have some effect on other health-related factors, including blood pressure, glucose metabolism, lipid profile, and liver enzymes.

### Fermented Dairy and Anti-inflammatory Immune Biomarkers

Four studies on fermented dairy examined biomarkers indicative of reductions in inflammation, encompassing the cytokines IL-10 and TGFβ (serum protein or transcript levels) and fecal metabolites, including secretory immunoglobulin A (sIgA), the antimicrobial peptide cathelicidin (LL-37), and α- and β-defensin (Table 2).

In a DB-CO-RCT, increased levels of IL-10 were detected in the serum of healthy female adolescents after consuming Greek yogurt daily during two 5-day training camps with intensified exercise, compared with when they consumed a nondairy control with isoenergetic carbohydrate levels (*N* = 13 subjects).^84^ The authors suggested that, although Greek yogurt was not associated with an added benefit in performance or muscle recovery, as determined by plasma creatine kinase and insulin-like growth factor-1 levels, it might aid in reducing inflammation by increasing plasma IL-10 during intensified training phases in young athletes (no differences in IL-6, TNFα, or CRP levels between groups). Another PG-RCT found that TGFβ gene transcript levels were significantly increased in peripheral blood mononuclear cells from healthy and obese patients who consumed yogurt over 9 weeks, compared with those who consumed soy pudding (*N* = 120 subjects).^85^ This study also reported a decrease in the TNF-α to soluble TNF II (sTNF-RII) ratio among those consuming yogurt, regardless of obesity. Lastly, increases in fecal sIgA, LL-37, and α- and β-defensins were found for children consuming milk fermented with probiotic *L paracasei* CBA L74, compared with those who received a nondairy control product in these 3-month studies.^66^^,^^67^ It is notable that these studies also found a reduced incidence of acute gastroenteritis, according to GI symptom surveys (Table 1). For one of those studies, there was a negative correlation between the change in anti-inflammatory biomarker levels and the number of CIDs reported for all participants.^67^

### Fermented Dairy and Intestinal Barrier Function

The intestinal epithelium is a selectively permeable, physical, and functional barrier, and therefore is an indicator of gut health.^86^^,^^87^ Intestinal barrier function can be measured using the Lactose/Mannitol (L/M) ratio test, which considers how mannitol, but not lactose, is selectively absorbed by the small intestine in healthy individuals.^88^^,^^89^ Barrier integrity can also be assessed by measuring epithelial cell tight-junction proteins and the presence of bacterial antigens (such as LPSs) in the serum.^87^ Four out of the 37 studies assessed the impact of fermented dairy products on biomarkers of intestinal barrier function (Table 1).

In the study with adults experiencing IBS-D,^57^ not only did the consumption of fermented milk result in reduced IBS scores, but there was also a reduced L/M ratio compared with baseline (Table 1).^57^ A CO-RCT trial with 28 overweight adults found that levels of serum zonulin, a protein that induces tight junction disassembly and increased intestinal permeability,^90^ were significantly lower after consuming kefir, but not milk, for 3 weeks.^91^ Similarly, obese women with MetS and NAFLD who consumed yogurt over 24 weeks and had reduced levels of serum TNFα also had decreased levels of serum LPS compared with those who consumed milk.^79^ Finally, a 9-week PG-RCT performed with 65 obese and 63 healthy women reported that low-fat yogurt intake was associated with a reduced ratio of serum LPS–binding protein (LBP) to soluble CD14 (sCD14) compared with subjects with an intake of soya (nondairy) pudding.^85^ A reduced LBP to sCD14 ratio is an indicator of an alleviation of endotoxemia.^92–94^ This result was consistent regardless of obesity status.^85^ Additionally, serum IgM EndoCAb (an antibody that is inversely associated with endotoxemia^95^^,^^96^) and 2-arachidonoylglycerol (a metabolite associated with the restoration of the intestinal barrier integrity^97^^,^^98^) levels were increased in obese subjects compared with baseline and the soya pudding control group, respectively.^85^ There were no significant changes in the levels of other biomarkers associated with barrier integrity disruption, such as IL-6, hs-CRP, or LPS, in that study.^85^

### Fermented Dairy and Gut Microbiota Composition and Fecal SCFA

A total of 10 studies assessed in this review evaluated the effect of fermented dairy on gut symptoms, immunity, and integrity and also measured the impact on the gut microbiota (Table 1). Another 5 studies focused solely on the effect of ferÍmented dairy consumption on the gut microbiota and/or SCFA (Table 1). Most of the investigations applied 16S rRNA gene amplicon DNA sequencing, although quantitative PCR (qPCR) and culture-dependent analyses were also used (Tables  1 and 3). The fecal microbiota composition was assessed using alpha- and beta-diversity metrics (Table 3) and comparisons of bacterial taxa (Table 4).

**Table 3.** Effect of Fermented Dairy Consumption on the Diversity of the Gut Microbiota and SCFA Levels

Study	Method^a^	Alpha and beta diversity changes after fermented dairy consumption		SCFA levels after fermented dairy consumption
		Alpha	Beta	
Volokh et al (2019)^104^	16S	**X**	**✓**	ND
Cannavale et al (2022)^105^	16S	**X**	**X**	ND
Guerin-Danan et al (1998)^107^	Plate culture	NA	NA	No effect
Canani et al (2017)^108^	16S	**X**	**X**	↑ Butyrate
Kato-Kataoka et al (2016)^43^	16S	**✓**	ND	ND
Liu et al (2010)^54^	Plate culture	NA	NA	NA
Le Nevé et al (2019)^58^	16S	ND	ND	ND
Le Nevé et al (2020)^45^	16S	**X**	**X**	ND
Zhang et al (2021)^47^	16S	**X**	**X**	ND
Guillemard et al (2021)^51^	16S	**X**	**✓**	↑ Butyrate, ↑ acetate, ↑ propionate, ↑ valerate
González et al (2019)^78^	qPCR	NA	NA	↑ Butyrate, ↑ acetate, ↑ propionate
Burton et al (2017)^81^	16S	ND	ND	ND
Bellikci-Koyu et al (2019)^80^	16S	**X**	**X**	ND
Chen et al (2019)^79^	16S	ND	ND	ND
Zheng et al (2015)^106^	ND	ND	ND	↑ Butyrate, ↑ propionate, ↑ malonate

a Fecal samples. Abbreviations: ↑, significant increase in levels of specified SCFAs; 16S, 16S rRNA gene amplicon sequencing analysis; **✓**, significantly changed diversity metric; **X**, no significant changes; NA, not applicable; ND, not determined because the study did not perform or report the analysis; SCFA, short-chain fatty acid.

**Table 4.** Responsive Intestinal Bacterial Taxa After Fermented Dairy Consumption

Taxonomic level^a^						Compared with baseline		Compared with placebo consumption	
Phylum	Class	Order	Family	Genus	Species	↑	↓	↑	↓
Actinomycetota								Bellikci-Koyu et al (2019)^80^	
	Actinomycetia	Bifidobacteriales	Bifidobacteriaceae	*Bifidobacterium*		Volokh et al (2019)^104^			Burton et al (2017)^81^
					*B kashiwanohense / B pseudocatenulatum*				Burton et al (2017)^81^
					*B adolescentis*			Cannavale et al (2022)^105^	
		Micrococcales	Micrococcaceae	*Rothia*					Zhang et al (2021)^47^
	Coriobacteriia	Eggerthellales	Eggerthellaceae	*Eggerthella*				Zhang et al (2021)^47^	
				*Adlercreutzia*		Zhang et al (2021)^47^			
					*A equolifaciens*	Volokh et al (2019)^104^			
				*Slackia*		Volokh et al (2019)^104^		Guillemard et al (2021)^51^†	
					*S isoflavoniconvertens*	Volokh et al (2019)^104^			
		Coriobacteriales	Coriobacteriaceae			Volokh et al (2019)^104^			
				*Collinsella*	*C aerofaciens*	Volokh et al (2019)^104^			
Bacteroidota	Bacteroidia	Bacteroidales	Bacteroidaceae			Volokh et al (2019)^104^			Kato-Kataoka et al (2016)^43^
				*Bacteroides*					González et al (2019)^78^
			Barnesiellaceae	*Coprobacter*					Guillemard et al (2021)^51^^†^
			Rikenellaceae RC9 gut group						Zhang et al (2021)^47^
Bacillota									Chen et al (2019)^79^
	Bacilli	Lactobacillales	Lactobacillaceae	*Lactobacillus*				Cannavale et al (2022)^105^, Canani et al (2017)^108^^c^	
					*L delbrueckii*			Burton et al (2017)^81^	
					*L zeae*			^105^	
			Streptococcaceae	*Streptococcus*	*S thermophilus*	Volokh et al (2019)^104^		Burton et al (2017)^81^	
		Bacillales	Staphylococcaceae			Volokh et al (2019)^104^			
	Clostridia								Chen et al (2019)^79^
		Clostridiales	Eubacteriaceae	*Eubacterium*	*E ventriosum* group				Chen et al (2019)^79^
		Eubacteriales	Lachnospiraceae	*Blautia*				Canani et al (2017)^108^c	Chen et al (2019)^79^
				*Pseudobutyrivibrio*					Chen et al (2019)^79^
				*Roseburia*			Volokh et al (2019)^104^, Guillemard et al (2021)^51^†	Canani et al (2017)^108^c	
				*Lachnoclostridium*/unclassified			Volokh et al (2019)^104^		
			Oscillospiraceae					Canani et al (2017)^108^c	
				*Faecalibacterium*				Canani et al (2017)^108^c	
				*Oscillibacter*					Zhang et al (2021)^47^
				*Ruminococcus*					Chen et al (2019)^79^
			Peptostreptococcaceae	*Intestinibacter*	*I bartlettii*			Burton et al (2017)^81^	
	Erysipelotrichia	Erysipelotrichales							Chen et al (2019)^79^
			Erysipelotrichaceae			Volokh et al (2019)^104^			Chen et al (2019)^79^
			Coprobacillaceae	*Catenibacterium*	*C mitsuokai*	Volokh et al (2019)^104^			
	Negativicutes							Chen et al (2019)^79^	
		Acidaminococcales	Acidaminococcaceae				Volokh et al (2019)^104^	Chen et al (2019)^79^	
		Selenomonadales						Chen et al (2019)^79^	
		Veillonellales	Veillonellaceae						Chen et al (2019)^79^
				*Dialister*			Guillemard et al (2021)^51^†		Chen et al (2019)^79^
				*Veillonella*		Zhang et al (2021)^47^			Burton et al (2017)^81^
				*Veillonella*				Zhang et al (2021)^47^	
Fusobacteriota	Fusobacteriia	Fusobacteriales	Fusobacteriaceae	*Fusobacterium*					Guillemard et al (2021)^51^†
Pseudomonadota	Betaproteobacteria	Burkholderiales	Burkholderiaceae	*Ralstonia*				Zhang et al (2021)^47^	
			Sutterellaceae	*Sutterella*					Zhang et al (2021)^47^
		Neisseriales	Neisseriaceae	*Neisseria*				Zhang et al (2021)^47^	
	Gammaproteobacteria	Enterobacterales	Enterobacteriaceae	*Escherichia-Shigella*					Guillemard et al (2021)^51^†
Thermodesulfobacteriota	Desulfovibrionia	Desulfovibrionales	Desulfovibrionaceae	*Bilophila*	*B wadsworthia*				Burton et al (2017)^81^
				*Desulfovibrio*				Guillemard et al (2021)^51^†	
Verrucomicrobiota	Verrucomicrobiia	Verrucomicrobiales	Akkermansiaceae	*Akkermansia*				González et al (2019)^78^	

a Taxonomy based on the deepest classification level.

b After 14-day *Helicobacter pylori* treatment and compared with control group.

c Genus classification based on oligotype. Abbreviations: ↑, significant increase; ↓, significant decrease.

Six studies measured gut symptoms and changes to the gut microbiota composition. Among those studies, 5 reported significant changes in both metrics after fermented dairy intake (Table 1). An increase in fecal bacterial alpha-diversity, number of species, and phylogenic diversity was reported in the PC-DB-PG-RCT with healthy medical students consuming milk fermented with the probiotic *L casei* Shirota YIT 9029 assessed for stress-induced abdominal dysfunction and GI symptoms.^43^ Patients with chronic liver disease who reported increased appetite and food intake, and decreased abdominal distension after consuming yogurt containing several undefined strains of “*B bifidum,” L acidophilus, L delbrueckii* subspecies *bulgaricus,* and *S thermophilus* for 14 days were also found to have reduced enteric bacilli (*E coli*) counts compared with baseline, according to bacterial enumeration from feces.^54^ In the PG-RCT study with IBS patients who reported reduced GI discomfort after consuming fermented milk containing the probiotic *B lactis* CNCM I-2494 twice daily for 14 days, compared with those consuming milk alone, the patients also had a decreased *Prevotella* to *Bacteroides* ratio.^58^ In the PC-DB-PG-RCT investigating the intake of probiotic *L paracasei* YIT 9029 (ie, strain Shirota) fermented milk among women with depression, it was found that both total patient constipation-symptom scores and serum IL-6 levels were reduced, and also that significant changes to the gut microbiota were induced.^47^ Fermented milk intake significantly reduced the relative abundance of Rikenellaceae_RC9_gut_group, *Sutterella*, and *Oscillibacter*, compared with milk consumption. The study did not detect changes in alpha- or beta-diversity.^47^ In the 4-week study assessing the benefits of probiotic fermented milk following *H pylori* eradication treatment, despite not finding improvements in gut symptoms or inflammatory biomarkers, it was found that there were significant decreases in intrasubject beta-diversity distance from baseline and decreases in proportions of *Escherichia-Shigella* and *Klebsiella* in the fecal samples of individuals who consumed the fermented milk compared with those from individuals who consumed milk.^51^ The study also reported increased fecal SCFAs in the fermented milk group compared with the control group.^51^ Lastly, no significant changes to the gut microbiota diversity were detected in an open-label study, which found reductions in gas-related symptoms to be associated with consumption of a fermented milk containing the probiotic *B lactis* CNCM I-2494.^45^ However, that study did note that the decreased number of anal gas evacuations and decreased sensation of flatulence associated with the consumption of fermented milk were correlated with a lower relative abundance of *Mogibacterium*, *Parvimonas*, *Methanobrevibacter* spp., and Cerasicoccaceae, and a higher relative abundance of Desulfovibrionaceae and *Succinivibrio*.^45^

Four studies on fermented dairy included measurements of biomarkers (immune, epithelial, and metabolism) and the gut microbiota (Table 1). In the cross-sectional trial that reported decreased serum levels of CRP among healthy individuals consuming diverse fermented dairy foods, it was found that intake of those foods was also associated with an altered fecal microbiota and increased fecal SCFAs, compared with individuals who did not report fermented dairy consumption.^78^ In that same study, according to qPCR targeting individual bacterial taxa, consumption of natural yogurt, but not sweetened yogurt, was associated with a significant increase in the fecal levels of *Akkermansia*, while sweetened yogurt reduced the levels of *Bacteroides*.^78^ Additionally, cheese intake was associated with increased acetate, propionate, and butyrate in the fecal contents.^78^ In the 2-week DB-CO-RCT with healthy young men who reported reduced serum TNFα, IL-6, and CCL5 levels compared with baseline levels with either acidified milk or probiotic-containing (*L rhamnosus* GG) yogurt consumption, modest reductions were also found in the proportions of the pathobiont *Bilophila wadsworthia*,^81^ a species associated with acute infection and inflammation.^102^^,^^103^ That study also reported that milk, but not yogurt, was associated with higher levels of intestinal *Bifidobacterium.*^81^ In the PC-PG-RCT wherein kefir intake was associated with reductions in serum TNFα and IFNγ,^80^ bacterial diversity did not change, but the proportions of Actinomycetota increased compared with baseline among kefir consumers. Anthropometric measurements and biochemical markers such as lipid profiles, inflammatory biomarkers, and blood pressure levels were positively correlated with the relative abundance of Bacillota, Proteobacteria, Actinomycetota, Porphyromonadaceae, and Veillonellaceae, and negatively correlated with the relative abundance of Bacteroidota and Clostridia.^80^ Additionally, the study wherein obese women with MetS and NAFLD were found to have reduced serum TNFα and LPS with regular yogurt consumption, also found them to have lower average proportions of bacteria in the Bacillota phylum and increased proportions of bacteria in the Negativicutes class.^79^

Three studies examined the effects of fermented dairy on the gut microbiota composition in adults, either without other comparisons^104^ or in relation to memory^105^ or metabolism. A 30-day open prospective controlled trial focused on the effects of probiotic yogurt (containing *B lactis* BB-12, but no other bacterial species or strain designations) on the gut microbiota of healthy volunteers (*N* = 150 subjects).^104^ Compared with baseline, there was a change in fecal bacteria beta-diversity and an increase in proportions of *Bifidobacterium* and the Coriobacteriia species *Adlercreutzia equolifaciens* and *Slackia isoﬂavoniconvertens*. Functionally, it was inferred from 16S rRNA gene sequence data that there was an increase in taxa with the ability to metabolize lactose and synthesize amino acids, alongside a decrease in the potential for LPS synthesis. Moreover, cluster analysis revealed that responders and nonresponders to probiotic yogurt–mediated alteration of the gut microbiota segregated into 2 groups based on their baseline microbial community structure.^104^ Another study (SB-CO-RCT; *N* = 26 subjects) examined the fecal microbiota of healthy adults after consuming either kefir (with 12 species of *Lactobacillus*, *Bifidobacterium*, and yeast [no strain designations or viable cell numbers were provided]) or lactose-free, low-fat milk intake for 4 weeks.^105^ No changes in the bacterial alpha or beta-diversity were found, but there were significant increases in the proportions of *Lactobacillus* in the stools of the individuals drinking kefir compared with milk.^105^ Notably, that study did find improvements in measures of relational memory performance, but there were no other improvements in symptoms of depression, anxiety, or stress, and no changes in urinary free-cortisol (a stress-related biomarker) concentrations were found.^105^ Improvements in relational memory performance were not correlated with changes in the microbiota. The third study reported on the impacts of cheese on blood cholesterol levels and the associated metabolic response, including SCFA levels.^106^ In that CO-RCT, 15 healthy young men consumed 3 isocaloric diets with similar fat contents—a diet high in milk, a diet high in cheese with equal amounts of dairy calcium, and a control diet—for 2 weeks. In addition to a potentially beneficial increase in lipid excretion, cheese consumption significantly increased fecal butyrate, propionate, and malonate, and urinary hippurate levels. These results suggest beneficial impacts of cheese, as compared to milk, on microbial and metabolic pathways in the intestine.

The remaining 2 studies assessed the impact of fermented dairy products on the gut microbiota in infants and children. For infants (*N* = 39 subjects) fed yogurt containing standard yogurt cultures (*S thermophilus* and *L delbrueckii* subspecies *bulgaricus*), fermented milk with the yogurt cultures and probiotic *L casei* DN-114 001, or milk daily for 1 month, yogurt consumption was associated with increased fecal enterococci levels, compared with baseline, according to culture-dependent analysis, and markers of proteolytic fermentation (branched-chain and long-chain fatty acids) decreased.^107^ Lactobacilli counts increased in the stools of infants given the fermented probiotic-containing milk, and β-glucuronidase and β-glucosidase activity was reduced. Otherwise, the numbers of total anaerobes (bifidobacteria, bacteroides, or enterobacteria) were not affected by fermented dairy consumption, nor were there significant changes in fecal SCFA quantities.^107^ Other changes in the intestinal microbiota were associated with the consumption of fermented milk with probiotic *L paracasei* CBA L74 for 3 months by children with CIDs.^108^ This study randomly selected 20 healthy children from a previous DB-PG-PC-RCT^67^ and reported that intake of fermented milk, as opposed to a maltodextrin drink (nondairy) with similar energy content, increased the relative abundance of *Lactobacillus*, Ruminococcaceae, *Roseburia*, *Blautia*, and *Faecalibacterium*, according to 16S rRNA gene amplicon sequence analysis.^108^ Supporting the increased levels of butyrate detected in fecal samples from subjects who consumed the fermented milk, these subjects also had significant changes to specific oligotype patterns of butyrate-producing bacteria, which included *Bacteroides*, *Blautia*, and *Roseburia*.^108^ The quantities of these bacteria were correlated with the anti-inflammatory immune markers LL-37, sIgA, β-defensin, and α-defensin.^108^

## DISCUSSION

**Figure 1.**
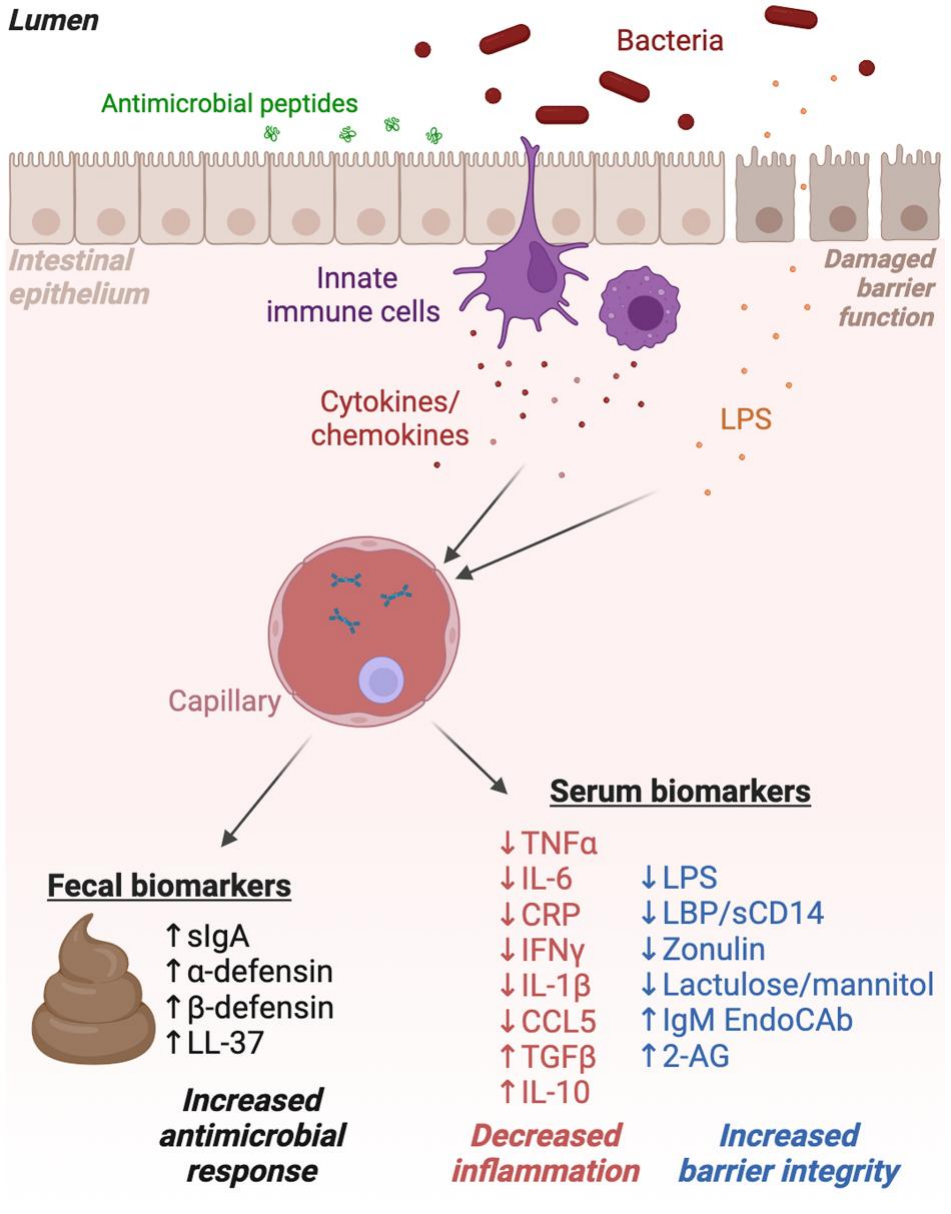
Impact of Fermented Dairy Consumption on Fecal and Serum Biomarkers of Inflammation and Intestinal Epithelial Barrier Integrity. Created in BioRender. Marco, M. (2025) https://BioRender.com/o70xunj. LPS, lipopolysaccharide

The importance of the GI tract in not only delivering basic nutrition but also influencing the risk of many metabolic, immune, and neurological diseases is becoming increasingly recognized and understood to be modulated by diet and the intestinal microbiome. Gut, or GI, health is a term that is frequently used to convey the overarching understanding of the connectivity between GI function and health. Although the generality of this concept presents challenges in interpretation and translation, assessment of clinical symptoms and quantification of intestinal and systemic biomarkers provide insight into the capacity of individual foods to affect GI function.^7^ Hence, we examined 37 human studies on fermented dairy foods—many of which contained documented probiotic strains—for their capacity to modulate metrics of gut health. These studies mainly showed that fermented dairy consumption by healthy adults or children or those experiencing underlying health conditions resulted in significant improvements in gut symptoms and biomarkers for gut immune responses and barrier integrity and changes to the distal (fecal) gut microbiota composition (Figure 1).

Fermented dairy, including yogurt, fermented milk, and kefir, was positively associated with improved symptoms of gut health in several RCTs across healthy adults and children. The studies found faster colonic transit times and improved stool frequency, appetite, and food intake, and reduced abdominal and rectal symptoms associated with discomfort or pain. Although dietary fiber has received more attention in human studies for its capacity to affect intestinal disorders,^18^ the findings of the studies discussed in this review indicate that fermented dairy products may be useful for individuals with an otherwise healthy lifestyle or diet who experience mild digestive manifestations. This is consistent with systematic reviews and meta-analyses of fermented dairy intake on immune and metabolic parameters.^27^^,^^70^^,^^109^ Although fermented dairy is better tolerated by lactose-intolerant individuals,^27^ future studies may improve understanding by differentiating between individuals based on their underlying sensitivity to lactose or dairy.

Fermented dairy intake had mixed effects on improving gut symptoms compared with regular milk consumption among adult IBS patients and patients undergoing treatment for *H pylori*. Although there is a potential for dairy to aggravate symptoms of IBS patients,^110^ fermented milk seems beneficial overall, although the modulation of specific symptoms is mixed. Variations in symptom changes may be due to the differences in the fermented milk products or the fact that IBS encompasses quite a broad range of GI symptoms. In this regard, fermented milk was typically more effective in alleviating GI symptoms among IBS patients with either predominate diarrhea or constipation as compared with those with more of a mixed profile.^56–61^ The 2 studies assessing the effects of fermented milk on patients undergoing *H pylori* therapy utilized different probiotics and intervention durations. Consumption of fermented milk with probiotic *B bifidum* YIT 4007 for 12 weeks improved GI symptoms,^50^ while a study with the multi-strain probiotic fermented milk (*L paracasei* CNCM I-1518 and I-3689 and *L rhamnosus* CNCM I-3690) found there was no effect.^51^

Fermented dairy consumption was associated with improvements in biomarkers of inflammatory responses, decreased and increased levels of proinflammatory- and anti-inflammatory cytokines, respectively, and with other proteins that modulate the intestinal barrier function. Prior meta-analyses reported that fermented dairy is not associated with a proinflammatory response.^109^^,^^111^ Instead, their findings suggest that fermented dairy-induced reductions in proinflammatory cytokines can potentially help prevent the development of more serious GI symptoms or diseases associated with high levels of inflammation.^109^^,^^111^ Reductions in TNFα levels were found in 6 of the 9 studies examined here in which TNFα levels were measured.^47^^,^^77^^,^^79–81^^,^^85^ In addition, 2 yogurt studies reporting reductions in TNFα levels also found lower levels of LPS.^79^^,^^85^ Fermented dairy may offer a potential benefit in enhancing the intestinal barrier, a finding consistent with a meta-analysis indicating benefits of probiotics for improving gut barrier function.^112^

In 14 out of the 15 studies for which the gut microbiota or SCFA were measured, fermented milk intake was associated with significant changes to the fecal microbiota composition. These findings were based on differences in the proportions of specific bacterial taxa (Table 3), altered alpha- or beta-bacterial diversity, modifications to fecal SCFAs (Table 4), or combinations of these metrics. However, the methods and taxonomic classification levels varied between studies, ranging from phylum to species-level descriptions. No specific taxon was consistently enriched across all trials, irrespective of whether the study compared fecal microbiota composition against a baseline time-point or placebo product. As shown recently, consumption of fermented dairy also impacts the microbiome in the small intestine,^113^ thus opening opportunities for investigating other potentially more direct fermented dairy–host interactions. With this noted, the clinical significance of using gut microbiota modulation as a biomarker for a change in gut health among individuals without GI infections remains uncertain.^114^ A healthy microbiome is difficult to define, and general changes in bacterial diversity or proportions of individual taxa may only be associative, rather than causal factors that result in changes to health.

Importantly, not all studies concluded that the consumption of fermented dairy resulted in benefits to gut health. These 10 studies examined different fermented dairy types, probiotic strains (if included), intervention durations, and study populations, and therefore there was no single condition or fermented dairy food that was found to be more or less effective than the others. No improvement was found in 3 of the 6 studies performed with the goal of improving symptoms of individuals with IBS.^59–61^ Only one of the 2 studies that were focused on the symptoms or inflammation status of patients with *H pylori* infections reported a benefit.^50^^,^^51^ For healthy adults, no benefit was found in 1 of 10 studies examining proinflammatory cytokines,^83^ and 1 out of 14 studies found no change in the gut microbiota composition.^45^ Lastly, 2 of the 5 studies performed with children and reporting on gut health symptoms found no benefit.^62^^,^^64^ Thus, until more studies are performed, it is not possible to generalize who would benefit most from fermented dairy consumption. Future research should identify optimal fermented dairy formulations and durations for different populations (eg, healthy adults, children, and individuals with specific diseases) to maximize gut-related benefits.

Despite the potential for fermented dairy foods to promote gastrointestinal health, several factors limit our capacity to reach a conclusion regarding the efficacy of fermented dairy consumption based on the 37 papers reviewed here. First, the 13 RCTs and observational study designs used different comparators (eg, compared with baseline or the placebo group), investigated both healthy individuals and those with diverse underlying conditions, and assessed different metrics of gut health. The duration of washout periods can also affect responses for cross-over RCTs, and other factors influencing generalizability include geographic and ethnic backgrounds.^115^ Variations in the fermented dairy product used and its microbial constituents are other factors that are expected to influence intervention outcomes. Yogurt, fermented milk, cheese, and kefir contain distinct ingredients and microbial consortia, resulting in differences in nutritional contents and bioactivity.^116^ The addition of probiotics to fermented dairy foods contributes another layer of complexity. Of the 37 studies, 19 studies included probiotic strains of either *Bifidobacterium* (7 studies), *Lactobacillus* (8 studies), or both (4 studies), which may produce distinct effector molecules with different mechanisms of action.^117^ Among the probiotics tested, one strain *B lactis* DN-173 010 (also named CNCM I-2494) was investigated in 6 RCTs that reported improved GI well-being, alleviated constipation by improving stool frequency and consistency, reduced IBS-C severity, and reduced gas-related symptoms in adults that consumed the fermented dairy product with this probiotic, but no effect was found in children with constipation.^42^^,^^45^^,^^46^^,^^56^^,^^58^^,^^62^ Lastly, the baseline intestinal microbiota may determine the responsiveness to fermented dairy intake. Two studies reported that the response to fermented dairy depended on the baseline gut microbiota composition.^58^^,^^104^ The gut microbiota was also previously found to significantly modulate host responses to other foods (eg, resistant starch) and therapeutic drugs.^118^^,^^119^ Therefore, more research is needed to fully understand the complex interactions occurring between an individual’s gut microbiota and habitual diet and the foods or nutrients modulated in dietary intervention studies.

These studies also would be greatly improved by a more thorough understanding of the mechanisms via which the microorganisms in fermented foods and their associated metabolites confer health benefits.^120^ For example, it is now well understood that yogurt starter cultures provide β-galactosidase, which continues to breakdown lactose after yogurt is consumed, thereby improving lactose tolerance in lactose-intolerant individuals.^121^ Yogurt also contains other metabolites, such as branched-chain hydroxy acids, which are associated with improved metabolic parameters in obese mice.^122^ By identifying the specific microorganisms and associated metabolites required for desired health benefits, “rationally designed” fermented dairy products can be produced. If safety is verified, these foods may also benefit the intestinal conditions experienced by critically ill individuals.^123^

In summary, the diversity of clinical and observational studies involving different designs, dairy products, intervention durations, and populations makes it challenging to draw definitive conclusions about the impact of fermented dairy on gut health. However, the findings suggest that yogurt, fermented milk, probiotic-fermented milk, and kefir consumption can alleviate GI symptoms and reduce inflammatory markers, particularly TNFα levels. Future investigations should prioritize efforts in assessments of the impact of well-defined fermented dairy foods on GI symptoms that are also associated with validated biomarkers for gut health. Ultimately, identifying fermented dairy food and strain combinations that affect specific metrics of gut health (eg, constipation or barrier function) will enable recommendations based on dietary and clinical needs.

## Data Availability

No new data were generated or analysed in support of this research.
